# Activation of PERK Signaling Attenuates Aβ-Mediated ER Stress

**DOI:** 10.1371/journal.pone.0010489

**Published:** 2010-05-05

**Authors:** Do Yeon Lee, Kyu-Sun Lee, Hyun Jung Lee, Do Hee Kim, Yoo Hun Noh, Kweon Yu, Hee-Yeon Jung, Sang Hyung Lee, Jun Young Lee, Young Chul Youn, Yoonhwa Jeong, Dae Kyong Kim, Won Bok Lee, Sung Su Kim

**Affiliations:** 1 Department of Anatomy and Cell Biology, College of Medicine, Chung-Ang University, Seoul, Korea; 2 Aging Research Center, Korea Research Institute of Bioscience and Biotechnology (KRIBB), Daejeon, Korea; 3 Department of Psychiatry, College of Medicine, Seoul National University, Seoul, Korea; 4 Department of Neurosurgery, College of Medicine, Seoul National University, Seoul, Korea; 5 Department of Neurology, College of Medicine, Chung-Ang University, Seoul, Korea; 6 Department of Food Science and Nutrition, College of Natural Science, Dankook University, Yongin, Korea; 7 Department of Environmental and Health Chemistry, College of Pharmacy, Chung-Ang University, Seoul, Korea; Brigham and Women's Hospital, Harvard Medical School, United States of America

## Abstract

Alzheimer's disease (AD) is characterized by the deposition of aggregated beta-amyloid (Aβ), which triggers a cellular stress response called the unfolded protein response (UPR). The UPR signaling pathway is a cellular defense system for dealing with the accumulation of misfolded proteins but switches to apoptosis when endoplasmic reticulum (ER) stress is prolonged. ER stress is involved in neurodegenerative diseases including AD, but the molecular mechanisms of ER stress-mediated Aβ neurotoxicity still remain unknown. Here, we show that treatment of Aβ triggers the UPR in the SK-N-SH human neuroblastoma cells. Aβ mediated UPR pathway accompanies the activation of protective pathways such as Grp78/Bip and PERK-eIF2α pathway, as well as the apoptotic pathways of the UPR such as CHOP and caspase-4. Knockdown of PERK enhances Aβ neurotoxicity through reducing the activation of eIF2α and Grp8/Bip in neurons. Salubrinal, an activator of the eIF2α pathway, significantly increased the Grp78/Bip ER chaperone resulted in attenuating caspase-4 dependent apoptosis in Aβ treated neurons. These results indicate that PERK-eIF2α pathway is a potential target for therapeutic applications in neurodegenerative diseases including AD.

## Introduction

Alzheimer's disease (AD), the most common form of dementia, is a chronic neurodegenerative disease causing progressive impairment of memory and other cognitive functions. Neuritic plaques, neurofibrillary tangles, and neuronal loss represent the main pathological characters in AD brains. Amyloid β-protein (Aβ), the central component of senile plaques, is produced from sequential proteolytic cleavages of the type 1 transmembrane β-amyloid precursor protein (APP) by β- and γ-secretase [1], [2]. Aggregated Aβ has been shown to interfere with several cellular processes and results the endoplasmic reticulum (ER) stress. ER stress triggers a cellular stress response called the unfolded protein response (UPR) intended to protect the cell against the toxic aggregated proteins [3].

The UPR is initiated by the binding of the ER chaperone GRP78/BiP to the misfolded proteins. Under normal conditions, GRP78/Bip sequester three key signal transducers at the ER membrane by forming the inactive complex; double-stranded RNA-activated protein kinase-like ER kinase (PERK), transcription factor ATF-6, and endoribonuclease IRE-1 [4], [5], [6]. Although the activation mechanisms of these ER-stress sensors are not fully understood, dissociation from GRP78/Bip seems to be required for the activation of three key signal transducers. One probable hypothesis is that the accumulating unfolded-protein preferentially binds GRP/Bip, which dissociates from PERK, ATF-6, and IRE-1. GRP78/Bip dissociation leads to autophosphorylation of PERK and IRE-1, and mobilization of ATF-6 to the Golgi for activation [7]. The activation of the UPR results in an overall decrease in translation, increased protein degradation and increased levels of ER chaperones, including GRP78/Bip [8], which consequently increases the protein folding capacity of the ER. Eventually, the cell might return to normal ER homeostasis or, under prolonged ER stress, continue towards apoptosis. As neurons are highly susceptible to the toxic effects of aggregated Aβ of AD, ER-stress-mediated cell death might have an important role in the pathogenesis of this disease [5]. Recently, several reports showed that the activation of UPR in neurons of AD brain [3] and oligomeric Aβ aggregates of Aβ1-42 peptide induce mild ER stress in neuronal cells [9]. Recent studies have demonstrated that activation of the UPR is a one of representative marker in both brain aging and age-related diseases of the brain. For example, the activation of the PERK pathway has been reported in the aged rodent models [10], [11], [12]. Similarly, studies have demonstrated in neurons of Alzheimer's disease [3] and in models of Parkinson's disease [13] that there is evidence for activation of the PERK pathway. Interestingly, phosphor-PERK stained neurons were overlapped with tau positive neurons [3], [14]. These data suggest that the PERK pathway is participated in the pathogenesis of aged related neurodegenerative diseases.

Activated PERK phosphorylates eukaryotic translation initiation factor 2 subunit α (eIF2α). After stress-induced phosphorylation of eIF2α, global protein translation of normal cellular mRNAs is repressed [15]. In parallel, translational initiation of transcription factor ATF4 is selectively stimulated. ATF4 induces the expression of downstream target genes such as GADD34, CHOP/GADD153 and others, which participate in the control of cellular redox status and cell death [12]. Importantly, the protein phosphates-1 (PP1) complex is inhibited by small molecule drug Salubrinal (Sal), which selectively blocks dephosphorylation of phoshpo-eIF2α [16]. Maintaining levels of p-eIF2α by Sal enhances cell survival in various cell lines against apoptosis induced by the ER stressors [8], [16].

However, the role of the UPR pathway, e.g. PERK signaling pathway, has not been elucidated in ER stress mediated Aβ neurotoxicity. Here, we demonstrate that the selective activation of PERK pathway is an early event of Aβ induced ER stress. PERK-eIF2α pathway promotes the induction of ER chaperones and confers resistant to aggregated protein toxicity in neuronal cells.

## Results

### Aβ activates UPR in SK-N-SH cells

**Figure 1:**
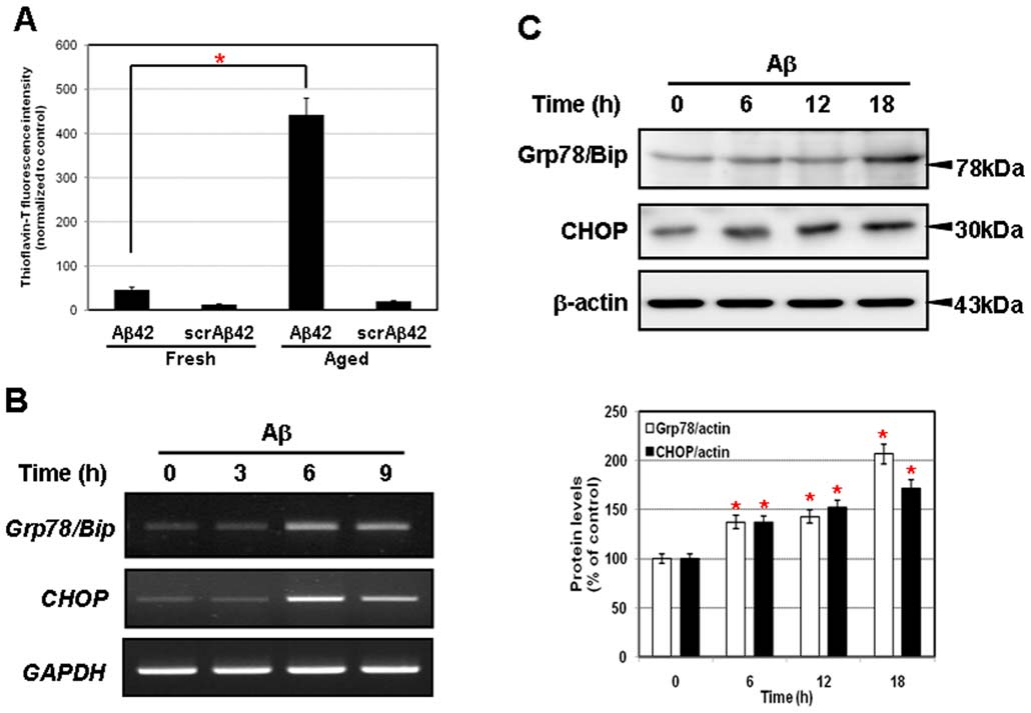
Aβ1-42 induces ER stress in SK-N-SH cells. **A**, Relative aggregation state of Aβ peptides were measured by thioflavin-T fluorometric assay in a cell-free system. Increased thioflavin-T fluorescence in aged Aβ42 peptide revealed greaterβ–sheet content in comparison with the fresh Aβ42 peptide but not in both fresh and aged of scrambled Aβ42 peptides (scrAβ42). Thioflavin-T fluorescence was monitored at 450 nm excitation and 482 nm emission. The expression levels of mRNA (**B**) and protein (**C**) of human Grp78/Bip, CHOP were increased in SK-N-SH cells treated with aged Aβ time dependent manner. GAPDH and β-actin were used as the loading controls. Data were presented as means ± SD from at least three independent experiments. ^*^ *P*<0.001.

Characterization of the aggregation status of Aβ42 is one of the critical issues in understanding the role of Aβ in the Alzheimer's disease. When acting on neuronal cells, whether it is the fibrillar or the non-fibrillar peptides shows different effect in neurotocixity. Reports from in vitro toxicity studies have suggested that aggregated Aβ is more toxic agent than soluble Aβ in cultured neurons [1], [17], [18]. In this study, we analyzed the effect of aggregation status of Aβ42 on UPR in neuronal cell. For that purpose, we prepared fresh and aged Aβ peptide solutions and their aggregation status were characterized by thioflavin-T (ThT) fluorescence. As shown in Fig. 1A, fluorescence intensity of aged Aβ42, prepared after incubation of the peptide solution for 7 days at 4°C, was significantly higher in comparing with fresh Aβ42. In contrast, the scrambled Aβ42 (scrAβ42) did not lead to any significant increase in the Th-T fluorescence levels (Fig. 1A).

To test whether UPR is activated in SK-N-SH human cholinergic neuroblastoma cells by aged Aβ42, we analyzed the levels of two known ER stress markers, Grp78/BiP and CHOP [19], using semi-quantitative RT-PCR and Western blot analysis. As shown in Fig. 1, Grp78/BiP and CHOP mRNA expression significantly up-regulated by aged 10 µM Aβ42 treatment from 6 h (Fig. 1B), consistent changes of protein levels were observed (Fig. 1C). On the contrary, the expression of Grp78/BiP and CHOP did not changed by treatment of fresh Aβ42 peptide and the scrAβ42 peptide. (Figure S1A). These results demonstrate that the aggregation status and the sequence of amino acids of Aβ peptide are critical for the activation of UPR in neuronal cells.

### Aβ preferentially induces PERK-eIF2a pathway

**Figure 2:**
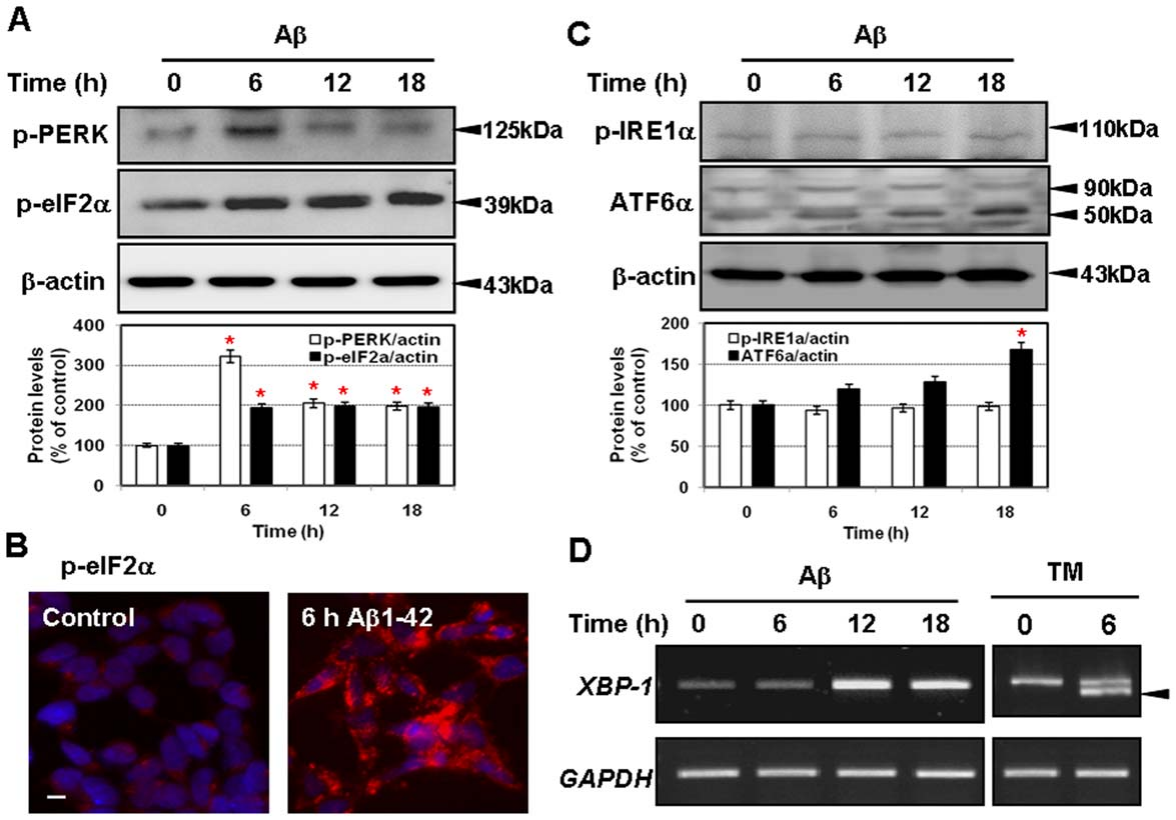
Aβ1-42 induces activation of PERK and eIF2α in SK-N-SH cells. **A**, Western blotting with anti-p-PERK (top) and anti-p-eIF2α (middle) in SK-N-SH cells treated with Aβ show the activation of PERK and eIF2α from 6 h. **B**, Immunostaining of p-eIF2α after 6 h Aβ treatment show the increased staining in cytoplasm. **C**, Western blotting with anti-p-IRE1α and anti-ATF-6 in SK-N-SH cells treated with Aβ. **D**, Unconventional splicing of the *XBP1* mRNA was not detected after Aβ treatment whereas Tunicamycin (TM; 2 µg/ml) treatment used as the positive control generated the spliced form of the *XBP1* mRNA. GAPDH and β-actin were used as the loading controls. Scale bar: 10 µm. ^*^ *P*<0.05.

To determine the activation status of three major ER stress sensors, PERK, IRE1α, and ATF6α, we performed the Western blot analysis with the antibodies against the phosphor-PERK, phosphor-IRE1α and ATF-6 in Aβ-treated neuronal cells. Interestingly, the levels of p-PERK and p-eIF2α in neurons were significant increased after 6 h by Aβ treatment (Fig. 2A). In contrast, the level of p-PERK and p-eIF2α did not changed by fresh Aβ42 peptide and the scrAβ42 peptide. (Figure S1B).

The activation of p-eIF2α was also detected in the immunostaining analysis. Staining intensity of cytoplasmic p-eIF2α level was significantly increased by Aβ treatment (Fig. 2B). These data showed that PERK-eIF2α pathway was definitely induced by Aβ in neuronal cells. In contrast, the activities of the other two UPR sensors, IRE1α and ATF6, did not changed in Aβ treated neuronal cells within 12 h (Fig. 2C). After 18 h, cleavage form of ATF6α was slightly increased by Aβ treatment but its induction was not as prominent as that of PERK-eIF2α (Fig. 2C). These results indicate that Aβ preferentially induces PERK-eIF2α pathway. The expression level of XBP-1 mRNA was increased by Aβ treatment but, unconventional splicing of XBP-1 mRNA, which is mediated by the endonuclease activity of IRE1α, was not detected in Aβ treated neuronal cell (Fig. 2D). These results indicate that the induction of ER stress by Aβ is not mediated via the IRE1-XBP1 pathway at least by 18 h after Aβ treatment. In summary, the activation of UPR in Aβ treated neuronal cells was detected as phosphorylation of PERK, p-eIF2α, and cleavage of ATF6. However, short-term treatment of Aβ (within 6 h) selectively augmented activation of the PERK pathway in neurons.

### Effects of PERK knockdown on Aβ-induced neuronal cell death

**Figure 3:**
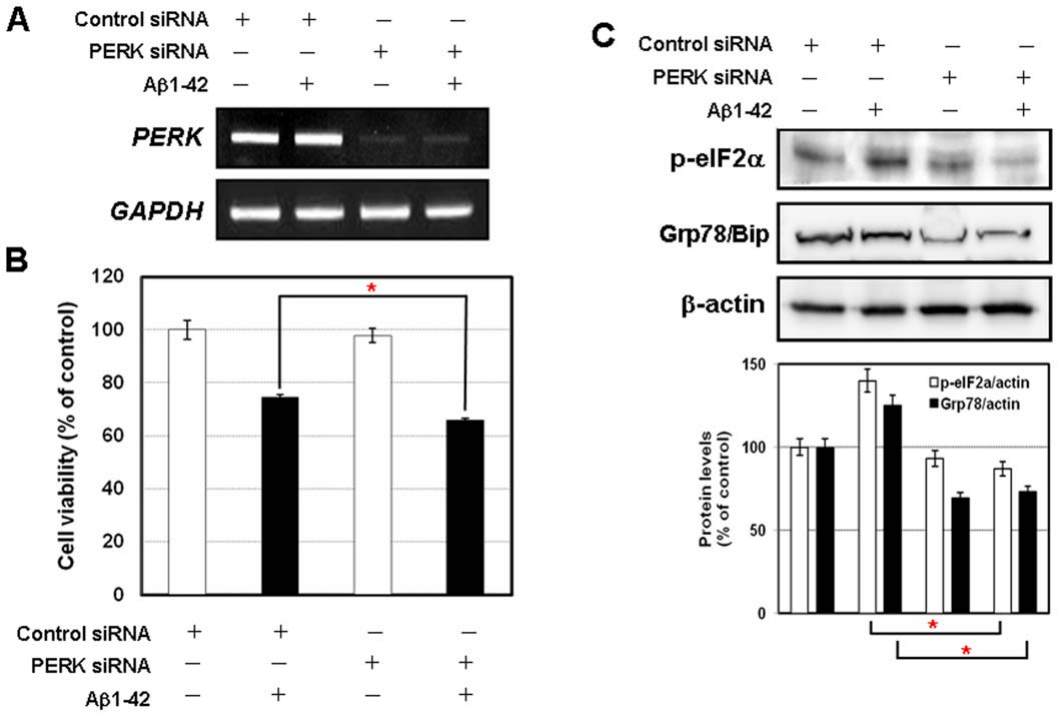
*PERK siRNA* reduces the neuronal cell viability by Aβ treatment. **A**, *PERK siRNA* transfection was confirmed by the *PERK* RT-PCR analysis. **B**, The cell viability in *PERK siRNA* transfection with Aβ treatment was reduced compared with Aβ treatment alone. Data were presented as means ± SD from at least three independent experiments. **C**, *PERK siRNA* transfection with Aβ treatment suppressed the activated eIF2α and Grp78/Bip by Aβ treatment alone. ^*^ *P*<0.05, control siRNA + Aβ versus PERK siRNA + Aβ.

To elucidate the role of PERK-eIF2α pathway in ER stress-mediated neuronal cell death by Aβ treatment, we knocked down expression of PERK by using siRNA against PERK. Transfection of PERK siRNA, but not control siRNA significantly reduced endogenous PERK mRNA levels (Fig. 3A). We then have assessed the role of PERK on ER stress mediated Aβ neurotoxicity. When treated with Aβ in SK-N-SH cells, silencing of PERK showed slightly enhanced cell death in comparison with those transfected with the control siRNA (Fig. 3B). These results indicate that PERK may play a role in cell survival mechanism rather than apoptosis on ER stress mediated Aβ neurotoxicity.

Next, we further tested whether knockdown of PERK abolishes eIF2α phosphorylation induced by Aβ. As expected, Aβ-induced eIF2α phosphorylation was significantly reduced in PERK knockdown cells (Fig. 3C). Grp78/Bip, ER-resident chaperone protein, is crucial for the modulation of UPR pathway under ER stress condition and functions as a cytoprotective protein in stressed cells [5]. Also, we tested the effect of siRNA silencing of PERK on the levels of Grp78/Bip. PERK knockdown significantly repressed the levels of Grp78/Bip induced by Aβ (Fig. 3C). These data indicate that PERK participates in the activation of p-eIF2α and Grp8/Bip in Aβ-mediated ER stress response in neuronal cells.

### Effects of Salubrinal, a selective activator of eIF2α, on Aβ-induced neuronal cell death

Salubrinal (Sal), a small molecule that protects cells from ER stress induced apoptosis by selectively activating an eIF2α branch of the UPR pathway [16]. When cells are challenged with ER stress, phosphorylated eIF2α is increased which mediates both a transient decrease in global translation and the translational up-regulation of selected stress-induced mRNAs. Phospho-eIF2α (p-eIF2α) is dephosphorylated by protein phosphatase-1 (PP1) complex. Importantly, the PP1 complex is inhibited by Sal, which selectively blocks dephosphorylation of p-eIF2α but not other PP1 substrates [16].

**Figure 4:**
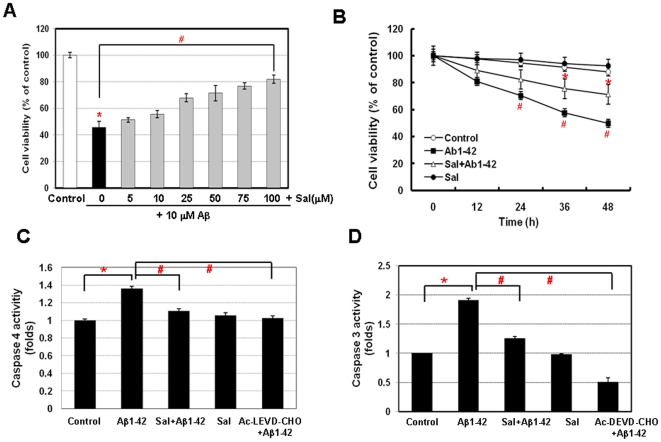
PERK activator Salubrinal attenuates Aβ1-42-induced neuronal apoptosis through the regulation of Grp78/Bip and caspase-4. **A**, Salubrinal protects neuronal cells against Aβ1-42-induced cell death. Dose-dependent protection by Salubrinal of SK-N-SH cells treated with Aβ and various concentrations of Salubrinal as indicated. **B**, Co-treatment of Salubrinal and Aβ increased the neuronal cell viability compared with Aβ treatment. Caspase-4 (**C**) and -3 activity (**D**) induced by Aβ was suppressed by the co-treatment of Salubrinal and Aβ. Data were presented as means ± SD from at least three independent experiments. ^*^ *P*<0.05, control versus vehicle alone; #*P*<0.05, control versus Aβ alone.

To investigate whether Sal has the ability to prevent neuronal apoptosis induced by Aβ, we treated various concentration of Sal for 2 h before Aβ treatment and assessed cell viability using alamarBlue assay. While cell viability was decreased by treatment of Aβ, pre-treatment with Sal significantly attenuated Aβ-induced neuronal cell death from 25 µM. Pre-treatment with 100 µM Sal reduced Aβ-induced neuronal cell death by 36.3±2.8% (Fig. 4A and Figure S2). In addition, Aβ-mediated cell death was significantly reduced by pre-treatment with 100 µM Sal compared to Aβ treatment alone from 24 h (Fig. 4B).

In humans, caspase-4, which was identified as the homologous gene to mouse caspase-12, has been shown to be specifically activated in ER stress-induced apoptosis and Aβ-induced neuronal cell death [20]. To determine whether Aβ-induced neuronal cell death required activation of apoptotic proteases, we measured the activities of caspase-4 and caspase-3. Aβ treatment increased caspase-4 activity by 1.4-fold, whereas pre-treatment with Sal reduced the caspase-4 activity to half of those in cells treated with Aβ only (Fig. 4C). This protective effect on neuronal cell death was also detected in the activity of caspase-3 (Fig. 4D) and the apoptotic morphological changes of nuclei (Figure S3).

### Effects of Salubrinal on UPR modulator Grp78/Bip

**Figure 5:**
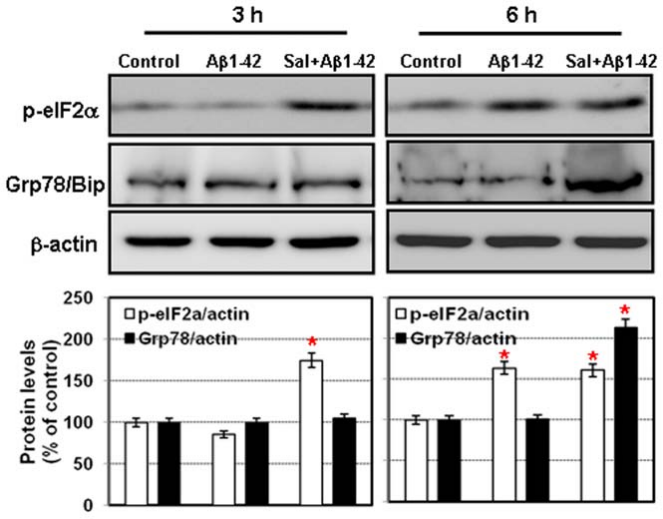
Western blot analyses with anti-p-eIF2α and anti-Grp78/Bip in SK-N-SH cells show that the co-treatment of Salubrinal and Aβ activated eIF2α at 3 h and Grp78/Bip at 6 h. β-acitn was used as the loading controls. ^*^ *P*<0.05.

Since PERK-eIF2α pathway may play a crucial role in cell survival rather than apoptosis in Aβ-induced neuronal cell death, we examined whether Sal affects on the induction of p-eIF2α and Grp78/Bip in SK-N-SH cells exposed to Aβ and/or Sal. As shown in Fig. 5, in the presence of Sal, Aβ increased eIF2α phophorylation from 3 h, whereas Aβ only increased eIF2α phophorylation after 6 h. In the absence of Sal, the level of Grp78/Bip expression was not changed by 6 h after Aβ treatment. In contrast, pre-treatment of Sal caused the highest induction of Grp78/Bip compared to Aβ alone. Taken together, these findings suggest that Sal, the selective activator of eIF2α, enhances Grp78/Bip expression in neuronal cells. Up-regulation of Grp78/Bip dependent PERK-eIF2a pathway seems to be a neuroprotective role against Aβ-induced neurotoxicity.

## Discussion

The results of this study are the first to show the protective effect of PERK pathway in Aβ mediated neuronal cell death. We show that treatment of Aβ1-42 triggers the UPR in the SK-N-SH human neuroblastoma cells. This event accompanies the activation of protective pathways of the UPR such as Grp78/Bip and PERK-eIF2α pathway, as well as the apoptotic pathways of the UPR such as CHOP and caspase-4. Previous studies reported that the induction of Grp78/Bip and phosphorylated PERK appeared in the brain of AD patients which is regarded as an early phenomenon in the pathology of AD [14]. ER stress specific caspase-4 is involved in neurotoxicity induced by Aβ25-35 and Aβ1-42 [20].

Mutations in the Presenilin genes PS-1 and PS-2 are the most common causes of early onset familial AD. It has been shown that these proteins are located predominantly within the ER [21]. The ER has been identified as the site where the highly toxic amyloidogenic Aβ peptide 1–42 is generated [22], and in brains manifesting sporadic AD, the ER-resident protein disulfide-isomerase (PDI) activity has been shown to be suppressed by S-nitrosylation [23]. Furthermore, PS-1 mutations linked to AD impair UPR signaling by inhibiting activation of PERK, IRE1 and ATF6 [24], [25]. Conversely, other studies have reported that phosphorylation of PERK and eIF2α has been found in neurons of AD, suggesting activation of UPR [14], [26].

During the last years, several evidences suggested that early intraneuronal accumulation of Aβ peptides is one of the key events leading to neuronal dysfunction in AD patients [27]. Synthetic Aβ1-40 and Aβ1-42 are amyloidogenic and neurotoxic peptides that have been widely used to mimic *in vitro* the degenerative process that occurs in the brain of AD patients [28]. Previous reports have been shown that extracellularly applied Aβ can be taken up by cultured neuronal cell lines [27], [29], [30] and co-localized in the endosomes/lysosomes or mitochondria [31], [32]. In addition, extracellularly treated Aβ aggregates induce mild ER stress in neuronal cells [9]. We thought that several mechanisms are involved in the Aβ-induced ER stress. Recently, Oh et al. [33] suggested that extracellular Aβ peptides enter the cell and inhibit the proteasome activity. Proteasome is the important machinery for ER-associated degradation (ERAD), which carries out eliminating the misfolded protein [34]. Another possible mechanism is the effect on calcium channel currents. Aβ affects calcium homeostasis by blocking Calcium influx at the plasma membrane or by perturbing Calcium storage in the ER [35], [36]. Therefore, it is possible that the proteasome dysfunction and the disturbance of calcium homeostasis result in the activation of UPR. The PERK-eIF2a pathway is the immediately early response among three UPR pathways, which leads to global translational attenuation [37]. These results suggest that the activation of PERK-eIF2a pathway induced by the internalized Aβ in the cytoplasm. Here we showed that the induction of eIF2α phosphorylation and Grp78/Bip by the treatment of eIF2α activator, Salubrianal, attenuated Aβ-induced neuronal cell death. These results suggests that PERK-eIF2α pathway is necessary for cell survival mechanism rather than cell death in this event, in agreement with results of other reports [16], [38]. Up-regulation of the ER chaperone protein Grp78/Bip has been recently noted in investigating the action mechanism of novel small molecules for diseases related to ER stress [8], [39]. Under conditions associated with ER stress, misfolded proteins accumulate in the ER lumen, a pathologic process resulting in the activation of the UPR pathway to combat the harmful effects of ER stress through the activation of ER chaperones such as Grp78/Bip. The Grp78/Bip was discovered as cellular proteins induced by glucose starvation [40]. Residing primarily in the ER, Grp78/Bip plays critical roles in the cellular stress of various diseases. In addition to facilitating proper protein folding, preventing intermediates from aggregating, and targeting misfolded protein for proteasome degradation, Grp78/Bip also binds Ca^2+^ and serves as an ER stress signaling regulator [40], [41]. Grp78/Bip is induced by ER stress for protecting against tissue or organ damage under pathologic conditions such as neurotoxic stress, myocardial infarction, and arteriosclerosis [41], [42]. Indeed, overexpression of ER chaperones such as Grp78/Bip, calnexin, and Grp170/Orp150 suppressed the production Aβ, a major component of extracellular senile plaques in AD [43].

Up-regulation of Grp78/Bip dependent PERK-eIF2α pathway is supposed to function as a neuroprotective role against Aβ neurotoxicity. In supporting this hypothesis, Sal, an activator of eIF2α of the UPR pathway, enhances Grp78/Bip expression for maintaining the normal ER homeostasis and cell viability. However, it is possible that Sal would impact differently on the other cellular signaling pathway. Additional further studies will be required in various cell system and animal models to understand fully the precise mechanism of Sal.

In summary, our results show that ER stress could be an important mechanism of early pathogenesis in Aβ1-42 induced neurotoxicity. In particular, the initial activation of the UPR mediated by PERK-eIF2α pathway might play a neuroprotective role to restore cellular homeostasis against Aβ-induced ER stress, thereby increase cell survival. On the basis of these data, we propose that the PERK-eIF2α pathway be part of the potential target for therapeutic applications in several neurodegenerative diseases related to pathological ER stress including AD.

## Materials and Methods

### Cell culture

SK-N-SH human neuroblastoma cells were obtained from American Type Culture Collection and cultured at 37°C in Dulbecco's Modified Eagle's Medium (DMEM) supplemented with 10% heat-inactivated FBS in a humidified 95% air, 5% CO_2_ incubator. Cell culture reagents were purchased from Gibco BRL. Salubrinal (Sal) and Tunicamycin (TM) were purchased from Tocris and Assay designs, respectively.

### Aβ1-42 preparation

The synthetic peptide Aβ1-42 (Aβ42) and scrambled Aβ1-42 (scrAβ42) were purchased from Biosource and rPeptide, respectively. The peptides were dissolved in D.W to 500 µM or in a diluted ammonia solution for facilitating peptide solubilization. Aβ1-42 or scrambled-Aβ42 aliquots were then store at −20°C until being used (fresh samples; Aβ42 or scrAβ42 peptide), or were incubated for 1 week at 4°C before use (aged samples; Aβ42 or scrAβ42 peptide).

### Aggregation state analysis of Aβ peptide

The fibril formation of Aβ peptides was measured by a thioflavin-T fluorometric assay as previously described with some modifications [44], [45], [46]. Fresh or aged Aβ peptide-containing samples were added to 3 µM thioflavin-T solution in a 50 mM glycine-NaOH buffer (pH 8.5). Fluorescence was measured at 450 nm excitation and 482 nm emission using a fluorescence spectrometer (Perkin-Elmer LS50). Each sample was determined in triplicates.

### Cell viability (alamarBlue assay)

For assessing apoptosis, alamarBlue assay was performed as described previously [47]. SK-N-SH cells were plated on 96-well plates (Nunc) at a density of 15,000 cells/well, in 100 µl of 10% FBS/MEM and incubated for 24 h. 2 h before 10 µM Aβ treatment, the media was replaced with 1% FBS/MEM. At the end of the treatment, 10 µl of alamarBlue agent (Serotec) was added. The cells were incubated for 3 h and then absorbance of the cells was measured at a wavelength of 570 nm using a microtiter plate spectrophotometer (FLUOstar Optima). The background absorbance was measured at 600 nm and subtracted. The cell viability was defined as [(test sample count)−(blank count)/(untreated control count)−(blank count)]×100.

### Hoechst 33258 staining

SK-N-SH cells were fixed with 4% paraformaldehyde for 20 min and then stained with 8 µg/ml of Hoechst dye 33258 (Sigma-Aldrich) for 5 min. They were washed twice with phosphate-buffered saline and observed using Axiovert 200 M equipped with ApoTom (Carl Zeiss). Dead cells and apoptotic bodies were characterized by condensed or fragmented nuclei.

### Caspase substrate cleavage assays

Caspase-3 and -4 activities were measured using colorimetric assay kits (BioVision Lab) as described previously [48]. Briefly, cells were collected and washed with ice-cold PBS and then resuspended in chilled lysis buffer for 20 min on ice. The supernatant was collected by centrifugation at 10,000 g for 5 min and assayed for protein content. For caspase activity measurements, cell extracts (20 µg protein) were incubated with 0.5 mM Ac-DEVD-*p*NA (caspase-3) or 0.5 mM Ac-LEVD-*p*NA (caspase-4) in a final volume of 100 µl at 37°C for 1 h. The release of the chromogenic compound *p*NA from the parent substrates was measured by absorbance at 405 nm using a microtiter plate spectrophotometer (FLUOstar Optima). Ac-DEVD-CHO (caspase-3) or Ac-LEVD–CHO (caspase-4) was used as a caspase-specific inhibitor (Sigma-Aldrich). Enzymatic activity is expressed as arbitrary units of relative value.

### RT-PCR analysis

Total RNA was isolated from the cells by using Trizol Reagent (Invitrogen) according to the manufacturer's instructions. cDNA was synthesized by using Superscript II Reverse Transcription system (Invitrogen). For RT-PCR, *AccuPower* PCR premix (Bioneer) was mixed with each primer. CHOP primers 5′-TTCTCTGGCTTGGCTGACTG-3′ (forward), 5′-CTGCGTATGTGGGATTGAGG-3′ (reverse); Grp78/Bip primers 5′-GCTCGACTCGAATTCCAAAG-3′ (forward), 5′-TTTGTCAGGGGTCTTTCACC-3′ (reverse); XBP-1 primers 5′-TAAGACAGCGCTTGGGGATC-3′ (forward), 5′-CTGGGGAAGGGCATTTGAAG-3′ (reverse); PERK primers 5′-ATCCCCCATGGAACGACCTG-3′ (forward), 5′-ACCCGCCAGGGACAAAAATG-3′ (reverse); GAPDH primers 5′- GGGGCTCTCCAGAACATCAT-3′ (forward), 5′-AAGTGGTCGTTGAGGGCAAT-3′ (reverse). Amplification conditions were as follows: single cycle of 94°C for 5 min followed by 30 cycles of 94°C for 30 s, 58°C for 30 s and 72°C for 30 s, and the final single cycle of 72°C extension for 7 min.

### Western blot analysis

Total proteins from SK-N-SH cells were isolated using the PROPREP protein extraction buffer (iNtRon biotechnology). Protein preparation and SDS-PAGE/immunoblotting were performed as previously described [48]. The cell homogenate was centrifuged at 1,000 g at 4°C for 10 min to discard unbroken or coarse cell debris and the resulting supernatant (RIPA lysate) was used for immunoblotting. Protein concentrations of RlPA lysates were determined by a modified Bradford method using BSA as a standard. Sample buffer (5% β-mercaptoethanol, 15% glycerol, 3% SDS, 0.1 M Tris, pH 6.8) was added to the aliquots (50 µg of protein) of the lysates, boiled for 3 min, and then resolved by 8∼12% SDS-polyacrylamide gel electophoresis (PAGE) under reducing conditions. The resolved proteins were transferred onto nitrocellulose membranes (Amersham Pharmacia Biotech, Littel Chalfont, UK) using a semidry trans-blot system (Schleicher & Schuell, Dassol, Germany). The blots were blocked for 2 h at room temperature with tris-buffered saline (TBS) (10 mM Tris, pH 7.5, 100 mM NaCl) containing 5% nonfat dry milk. The blots were washed three times with TBS, and then incubated at room temperature overnight with Anti-KDEL (1∶1000, Assay designs), anti-GADD153/CHOP (1∶1000, Santa Cruz Biotechnology), anti-phospho-PERK (1∶800, Santa Cruz Biotechnology), anti-phospho-eIF2α (1∶1000, Cell Signaling), anti-phospho-Ire1α from (1∶800, Abcam), anti-ATF6α (1∶1000, Santa Cruz Biotechnology), or β-actin (1∶2000, Abcam) were used for primary antibodies in TBST (10 mM Tris, pH 7.5, 100 mM NaCl, 0.05% Tween 20) containing 1% nonfat dry milk. The next day, the blots were washed three times with TBST, and then incubated for 1 h at room temperature with horseradish peroxidase (HRP)-conjugated secondary antibodies (1∶2000 dilution) (Santa Cruz Biotechology) in TBST containing 1% nonfat dry milk. After washing three times with TBST, the protein was visualized using the ECL detection system (Amersham Pharmacia Biotech).

### Immunocytochemisty

Immunostaining was performed as described previously [49]. Cells grown on glass cover slides, were washed with PBS and fixed in 10% formalin solution containing 4% formaldehyde for 20 min at RT, then incubated with Phospho-eIF2α (1∶1000, Cell Signaling) primary antibody, revealed with anti-rabbit IgG Alexa 594 secondary antibody (1∶200, Molecular Probe). After reaction with secondary antibodies, the cells were stained with 100 nM DAPI (4′,6-diamidino-2-phenylindole)(Molecular Probes) for 5 min, and mounted. Fluorescence-labeled cells were visualized using Axiovert 200 M equipped with ApoTom (Carl Zeiss).

### Small interference RNA (siRNA) for PERK

SK-N-SH cells were seeded onto 6-well plates and allowed to reach 50% confluence on the day of transfection. The small interfering RNA (siRNA) constructs used were obtained as the siGENOME SMARTpool reagents (Dharmacon), the siGENOME SMARTpool PERK (M-004883-03-0020). The non-targeting siRNA control, SiConTRol non-targeting SiRNA pool (D-001206-13-20) was also obtained from Dharmacon. Cells were transfected with 100 nM siRNA diluted in Opti-Eagle's Minimal Essential Medium (MEM) using Lipofectamine reagent (Invitrogen) according to the manufacturer's transfection protocol.

### Statistical analysis

All data are expressed as the means ± SD. To determine the significance of differences between the means of two groups, an unpaired two-tailed Student's *t*-test was applied to study the relationship between the different variables. To determine the significance of differences among the means of several groups, one-way analysis of variance (ANOVA) followed by Scheffe's post-hoc tests were applied. Statistical significance was determined via ANOVA followed by Scheffe's post-hoc tests. A p-value of <0.05 was considered to be significant.

## Supporting Information

Figure S1 Aged Aβ1-42 peptide induces ER stress and activation of PERK-eIF2α in SK-N-SH cells. Cells were treated with Aβ42 peptide or scrambled Aβ42 peptide (scrAβ42) in fresh or aged condition. A, The expression levels of protein of human Grp78/Bip (top) and CHOP (middle) were increased in SK-N-SH cells treated with aged Aβ42 but not fresh Aβ42 at 18 h. B, Western blotting with anti-p-PERK (top) and anti-p-eIF2α (middle) in SK-N-SH cells treated with aged Aβ42 show the activation of PERK and eIF2α from 6 h but not fresh Aβ42. The scrambled Aβ42 peptides (scrAβ42) did not lead to any significant increase in both fresh and aged condition, demonstrating that the specific sequence of amino acids of Aβ peptide is needed for the induction of ER stress and the activation of PERK-eIF2α. β-actin was used as the loading control (bottom).
(2.23 MB TIF)

Figure S2 Effects of Salubrinal on cell viability in SK-N-SH cells. Cells were treated with various concentrations of Salubrinal as indicated. Tunicamycin (TM, 2 µg/ml) was used as the positive control. Cell viability was measured by alamarBlue assay from 48 h after each treatment. *P<0.01, versus control (vehicle alone).
(1.18 MB TIF)

Figure S3 Salubrinal inhibits Aβ1-42-induced neuronal apoptosis. Cells were stained with Hoechst 33258 staining. Dead cells were identified by morphological changes, such as nuclei fragmentation (arrowhead), compared with normal cell nuclei. Scale bar: 10 µm.
(2.50 MB TIF)
