# Supplementary figures and images for: Activation of PERK Signaling Attenuates Aβ-Mediated ER Stress
Source: PLoS One. 2010 May 5;5(5):e10489. doi: 10.1371/journal.pone.0010489 (PMC2864758; doi:10.1371/journal.pone.0010489)

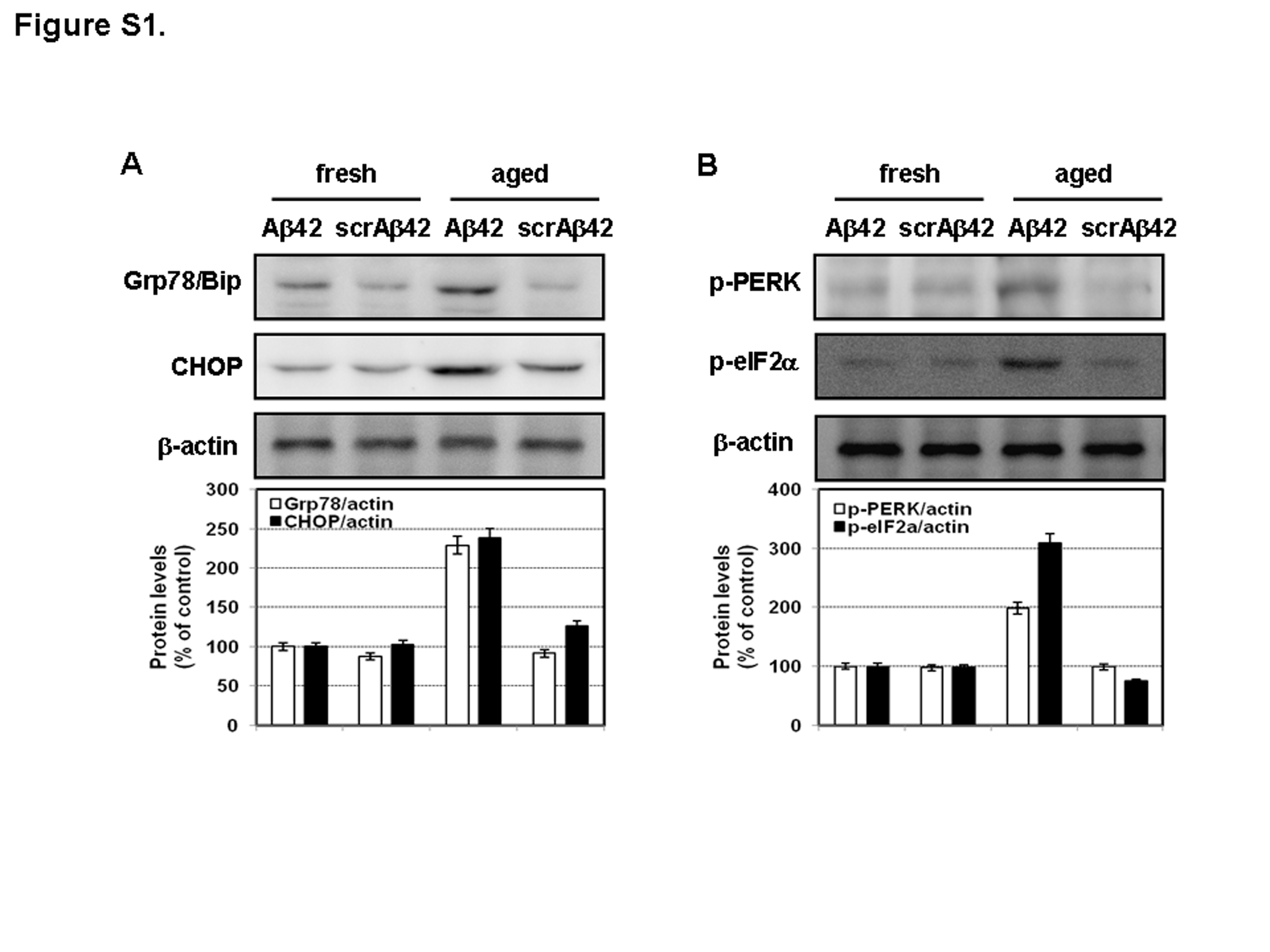

Supplement: Figure S1 — Aged Aβ1-42 peptide induces ER stress and activation of PERK-eIF2α in SK-N-SH cells. Cells were treated with Aβ42 peptide or scrambled Aβ42 peptide (scrAβ42) in fresh or aged condition. A, The expression levels of protein of human Grp78/Bip (top) and CHOP (middle) were increased in SK-N-SH cells treated with aged Aβ42 but not fresh Aβ42 at 18 h. B, Western blotting with anti-p-PERK (top) and anti-p-eIF2α (middle) in SK-N-SH cells treated with aged Aβ42 show the activation of PERK and eIF2α from 6 h but not fresh Aβ42. The scrambled Aβ42 peptides (scrAβ42) did not lead to any significant increase in both fresh and aged condition, demonstrating that the specific sequence of amino acids of Aβ peptide is needed for the induction of ER stress and the activation of PERK-eIF2α. β-actin was used as the loading control (bottom). (2.23 MB TIF) [file pone.0010489.s001.tif]

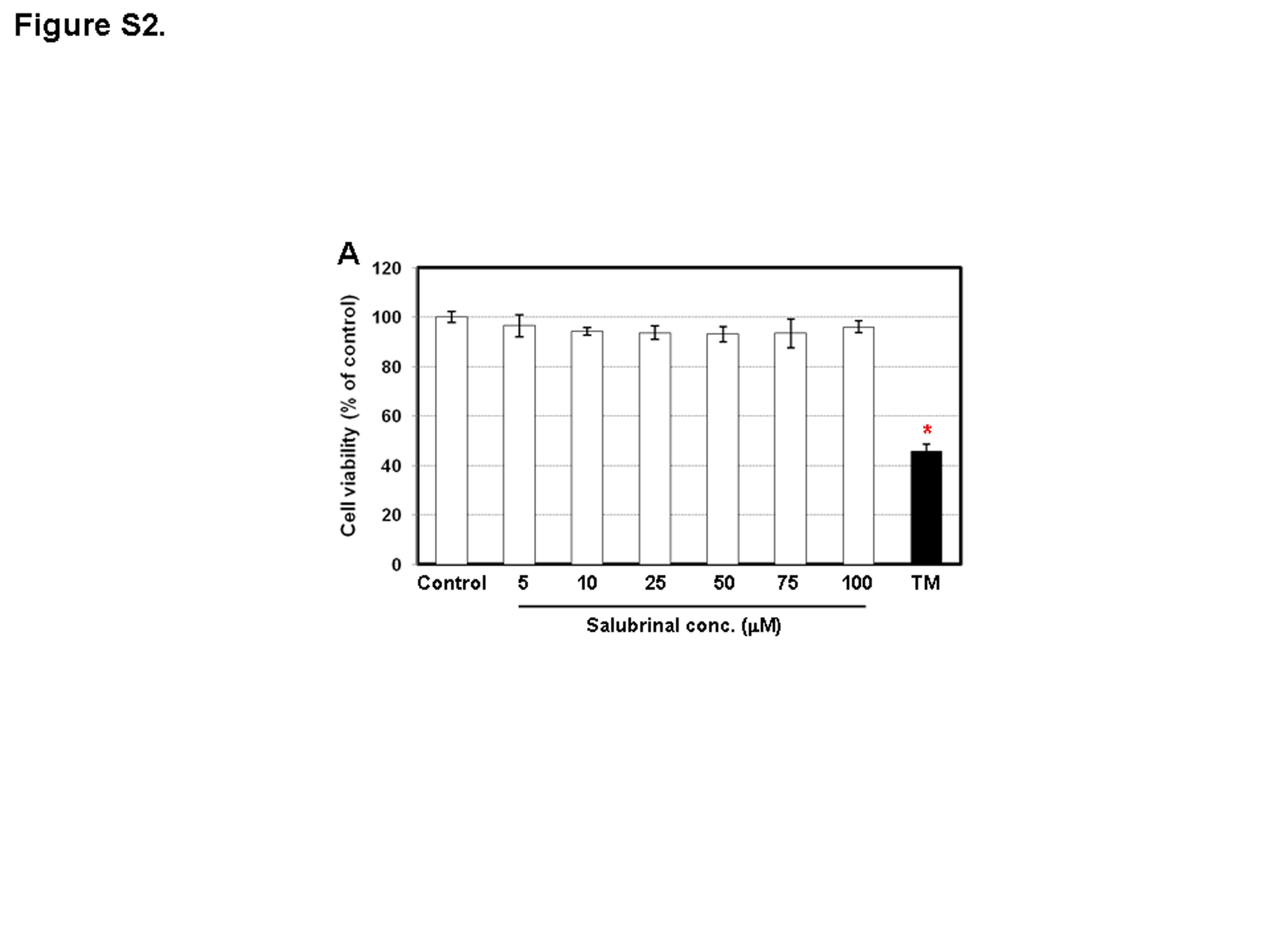

Supplement: Figure S2 — Effects of Salubrinal on cell viability in SK-N-SH cells. Cells were treated with various concentrations of Salubrinal as indicated. Tunicamycin (TM, 2 µg/ml) was used as the positive control. Cell viability was measured by alamarBlue assay from 48 h after each treatment. *P<0.01, versus control (vehicle alone). (1.18 MB TIF) [file pone.0010489.s002.tif]

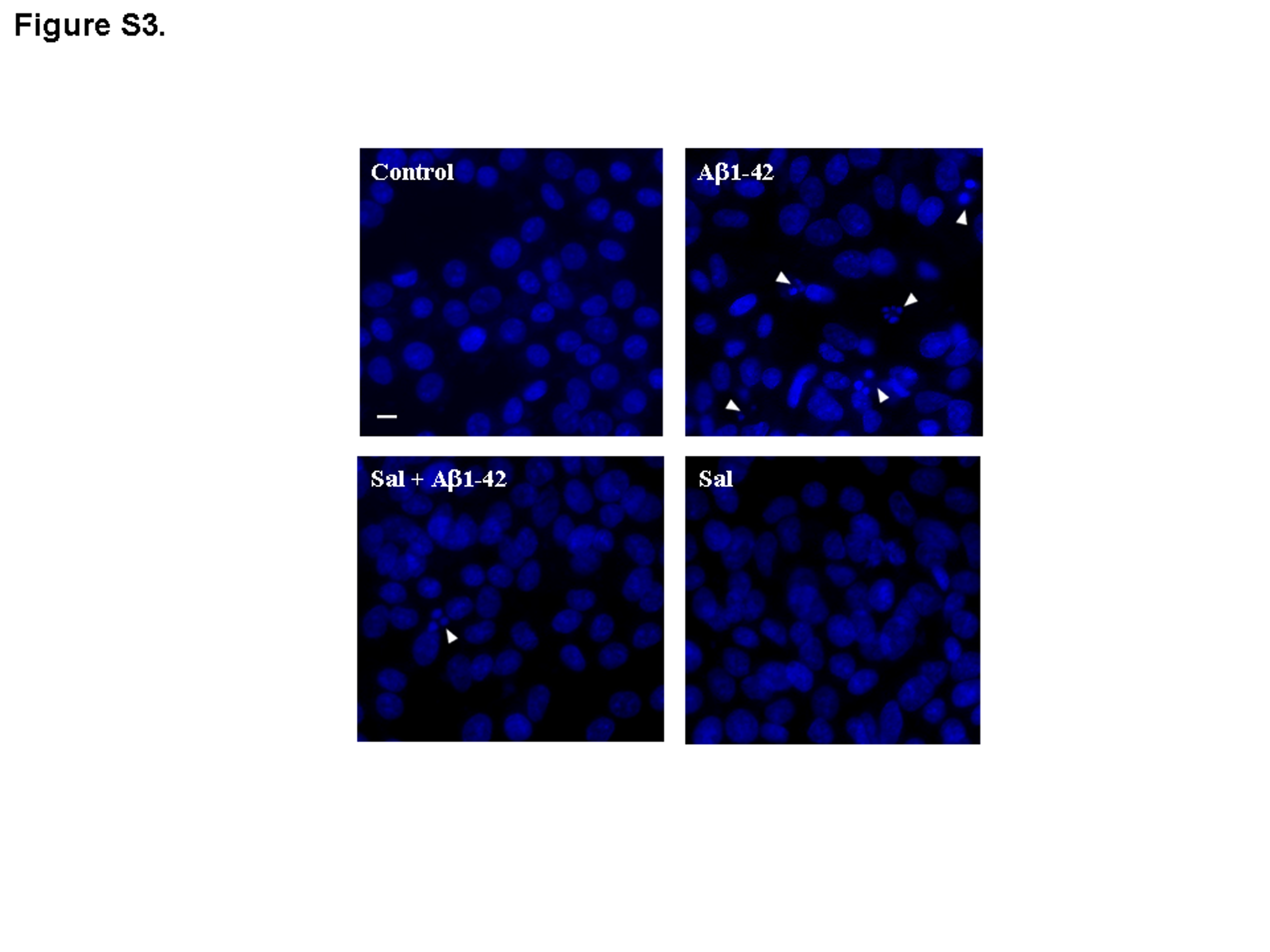

Supplement: Figure S3 — Salubrinal inhibits Aβ1-42-induced neuronal apoptosis. Cells were stained with Hoechst 33258 staining. Dead cells were identified by morphological changes, such as nuclei fragmentation (arrowhead), compared with normal cell nuclei. Scale bar: 10 µm. (2.50 MB TIF) [file pone.0010489.s003.tif]
